# Tel-eVax: a genetic vaccine targeting telomerase for treatment of canine lymphoma

**DOI:** 10.1186/s12967-018-1738-6

**Published:** 2018-12-11

**Authors:** Joseph A. Impellizeri, Alessandra Gavazza, Eliana Greissworth, Anna Crispo, Maurizio Montella, Gennaro Ciliberto, George Lubas, Luigi Aurisicchio

**Affiliations:** 1 Veterinary Oncology Services, PLLC, New York, NY USA; 2 Dept. of Veterinary Clinics, University of Pisa, Pisa, Italy; 3 Istituto Nazionale Tumori “Pascale”, Naples, Italy; 4 Istituto Nazionale Tumori “Regina Elena”, Rome, Italy; 5 BIOGEM Scarl, via Camporeale, 83031 Ariano Irpino, AV Italy; 6 Evvivax s.r.l., via di Castel Romano 100, 00128 Rome, Italy

**Keywords:** Cancer vaccine, Canine lymphoma, Genetic vaccine, TERT

## Abstract

**Background:**

we have recently shown that Tel-eVax, a genetic vaccine targeting dog telomerase (dTERT) and based on Adenovirus (Ad)/DNA Electro-Gene-Transfer (DNA–EGT) technology can induce strong immune response and increase overall survival (OS) of dogs affected by multicentric Diffuse Large B cell Lymphoma (DLBCL) when combined to COP therapy in a double-arm study. Here, we have utilized a clinically validated device for veterinary electroporation called Vet-ePorator™, based on Cliniporator™ technology currently utilized and approved in Europe for electrochemotherapy applications and adapted to electrogenetransfer (EGT).

**Methods:**

17 dogs affected by DLBCL were vaccinated using two Ad vector injections (Prime phase) followed by DNA–EGT (Boost phase) by means of a Vet-ePorator™ device and treated in the same time with a 27-week Madison Wisconsin CHOP protocol. The immune response was measured by ELISA assays using pool of peptides.

**Results:**

No significant adverse effects were observed. The OS of vaccine/CHOP animals was 64.5 weeks, in line with the previous study. Dogs developed antibodies against the immunizing antigen.

**Conclusions:**

Tel-eVax in combination with CHOP is safe and immunogenic in lymphoma canine patients. These data confirm the therapeutic efficacy of dTERT vaccine and hold promise for the treatment of dogs affected by other cancer types. More importantly, our findings may translate to human clinical trials and represent new strategies for cancer treatment.

## Background

Lymphoma is one of the most common malignancies diagnosed in pet dogs in the United States [1, 2]. Among these canine lymphoma accounts for up to 24% of all reported neoplasms and the majority (60–80%) arises from malignant B cells. The most common presentation is a generalized multicentric lymphadenopathy corresponding from stage III to V of the disease, with stage V describing tumor in blood, bone marrow, and other organ systems [3]. The current standard-of-care for canine Diffuse Large B-cell Lymphoma (DLBCL) is the combination chemotherapy regimen of cyclophosphamide, vincristine and prednisolone (COP) or cyclophosphamide, vincristine, doxorubicin, and prednisone (CHOP) regimen with or without l-asparaginase [4–7]. Negative prognostic factors, including, but not limited to, sub-stage b, T-cell immunophenotype, chronic inflammation, advanced stage and high body weight can reduce this expected survival time significantly [1, 2, 8–13]. A recent study analysed the survival data in 598 dogs and showed geographic differences in the first remission durations of pet dogs diagnosed and treated at referral institutions with CHOP chemotherapy in the continental United States [14]. The median PFS for all B cell-LSA was 234 days (interquartile range 109–343 days) and was in line with other studies [4, 15, 16]. In the northern United States (Connecticut, Illinois, Indiana, Iowa, Massachusetts, Michigan, New Hampshire, New Jersey, New York, Pennsylvania, Rhode Island and Wisconsin), the median PFS was 244 days (interquartile range 109–343 days).

In humans, DLBCL is the most common high-grade non-Hodgkin lymphoma. Immunochemotherapy with rituximab and CHOP (R-CHOP) is the current first-line treatment [17]. However, with this therapeutic approach up to 40% of patients experience early treatment failure or relapse after initial response [18]. Table 1 reports a side-by-side comparison and the current therapeutic approaches for human [19, 20] and canine DLBCL [21]. Considering the average life span of dogs and humans (about sevenfold difference), this further confirms the high translational relevance of canine patients in Comparative Oncology initiatives.

**Table 1:** comparison between human and canine DLBCL

Species	Median age of occurrence	Molecular biomarkers	Current treatment	PFS/OS
Human	≥ 65 years [19]	Myc, BCL2, BCL6 [20]	R-CHOP [17]	> 8 years [19]
Dog	7 years [12]	Myc, BCL2 [21]	COP/CHOP	0.7 years

Telomerase is a ribonucleoprotein comprising an RNA component and a catalytic protein component (telomerase reverse transcriptase, TERT) [22, 23]. As reported for other species, telomerase activity has been observed in the majority (> 90%) of canine tumors [24] contributing to maintenance of telomere length in cancer cells. Telomerase expression and telomere maintenance are critical for cell proliferation and survival in hematological malignancies [25].

Genetic vaccines are emerging among the most promising methodologies in cancer treatment. Evidence points towards the genetic immunization modality (*heterologous* prime/boost) as a powerful approach to induce superior immune responses and achieve greater clinical efficacy [26–30], including veterinary [31] and translational oncology [32].

We have recently shown that Tel-eVax, a genetic vaccine based on Adenovirus (Ad) and DNA Electro-Gene-Transfer (DNA–EGT) and targeting dTERT was able to induce strong immune response in dogs affected by B-cell LSA. Most importantly, COP regimen did not interfere with the effects of the immunotherapy and the survival of canine lymphoma was significantly augmented in comparison to chemotherapy treated subjects in two different studies [33, 34].

In this study, we evaluated the impact of Tel-eVax in association with CHOP chemotherapy. In addition, we have tested the presence of anti-TERT antibodies as potential surrogate efficacy biomarkers.

## Methods

### Study design

From September 2010 to July 2017, 45 client-owned dogs with multicentric DLBCL were evaluated and 17 enrolled in the study. For each dog, the veterinary staff of the Veterinary Oncology Services (VOS) performed a full initial clinical examination and administered both the chemotherapy and the immunotherapy. Every patient had a histopathologic or cytologic diagnosis of DLBCL (using the immunophenotype assessment) and an informed consent was distributed and approved by the owners.

### Blood tests

Complete blood count, serum biochemical profile of at least 10–12 analytes (including total protein, albumin, urea, creatinine, alanine aminotransferase, aspartate aminotransferase, alkaline phosphatase, gamma-glutamyltransferase, calcium, phosphorus, iron, cholesterol, glucose, sodium, potassium in serum) and urinalysis were obtained from each patient. When elected by the owner, full staging was also performed.

### Dogs immunization

The genetic components of Tel-eVax (Ad6-dTERT_opt_ and pV1J-dTERT.LTB_opt_) have been described elsewhere [35]. Briefly, Ad6-dTERT_opt_ expresses a codon optimized, catalytically inactive (D702A, V703I) full length canine telomerase and pV1J-dTERT.LTB_opt_ encodes the same cDNA fused at N-term with tissue plasminogen activator (TPA) leader sequence and at the C-term with the codon optimized B subunit of *E. coli* heat-labile enterotoxin (LTB). For Ad vaccination (prime), dogs were injected in the biceps femoris muscle with a dose of 10^11^ Ad viral particles (vp). The DNA injection (boost) consisted of a 1 ml solution (split over two injection sites with 0.5 ml/site) containing 5 mg pV1J-dTERT.LTB_opt_ in the dogs’ *tibialis cranialis* muscle. Dogs received two Ad6-dTERT_opt_ injections followed by one or more cycles, each composed of 3× DNA–EGT. The electroporation was carried out with Vet-ePorator™, a device manufactured by IGEA (Carpi, Modena, Italy) for Evvivax and based on Cliniporator^®^ Technology. Electrical conditions consisted of 8 square unipolar pulses at 110 V, at an interval of 120 ms. The pulse length was 20 ms/phase with a frequency of 8 Hz. The total treatment lasts less than 10 s. Dogs were anesthetized to carry out the electroporation with propofol induction and either Isofluorane or Sevofluorane inhalant anesthesia with oxygen. The entire electroporation procedure, including anesthesia, lasted for about 10 min. Blood was collected at indicated time-points and serum was frozen for further analysis.

### Peptides

Lyophilized dTERT peptides were purchased from JPT Peptide Technologies GmbH (Berlin, Germany) and resuspended in dimethyl sulfoxide (DMSO) at 40 mg/ml. Pools of 15 amino acid peptides overlapping by 11 residues were assembled as described previously [36]. Four pools of 70 peptides (A, B, C and D) were formed for the whole length of TERT protein. The final concentration of each peptide in the pools was 0.57 mg/ml.

### ELISA assay

Antibodies against TERT were measured by ELISA. 96 well plates were coated with peptide pools diluted at 1 μg/ml at overnight at 4 °C in coating buffer (carbonate buffer 50 mM pH 9.6). After washing with PBS and blocking with 5%BSA PBS-Tween 0.05%, sera from DNA–EGT immunized animals were incubated O/N at 4 °C at different dilution and revealed by a secondary antibody, AP-conjugated goat anti-dog IgG (SIGMA A0793, whole molecule) at 1:2000 in 1%BSA PBS-Tween 0.05% and incubated for 30′ at RT. Plates were read on an ELISA reader at OD_405–620_. Optical density values were plotted.

### Statistical analysis

Student’s t-test was performed where indicated. Log-rank test was used to evaluate the difference between overall survival (OS) between groups.

## Results

### Study design and Patients

**Fig. 1:**
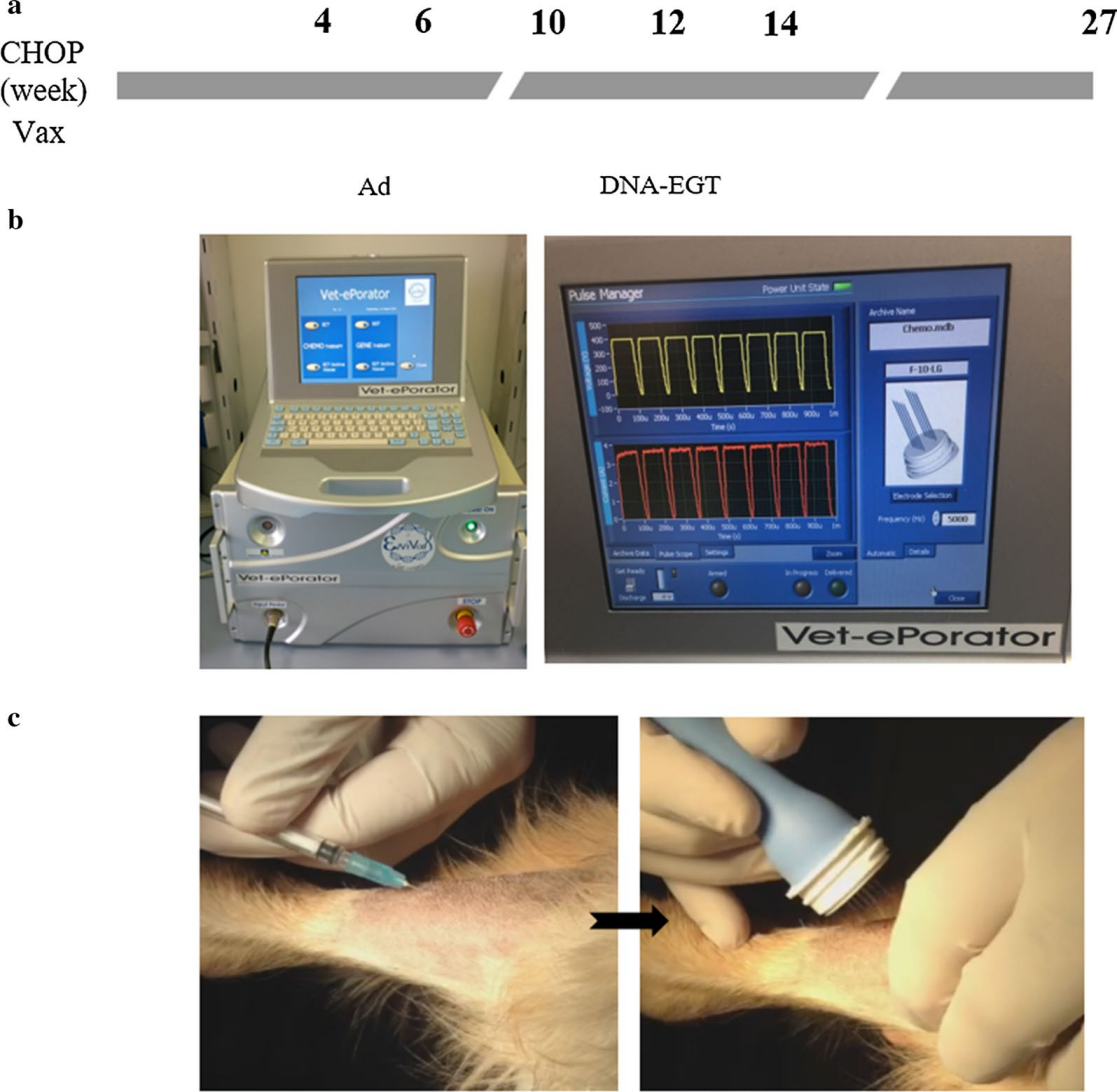
Vaccination procedure. **a** Vaccination schedule. Dogs received a l-CHOP 27-weeks protocol and vaccine was administered at week 4, 6 (Ad, triangles), 10, 12 and 14 (DNA–EGT, arrows). **b** Vet-ePorator™ device. Electroporation data are visualized in real time during pulse delivery. **c** DNA injection procedure. *Tibialis cranialis* muscle is injected with DNA, followed by electrodes insertion and application of electric field

A total of 17, stage III–V dogs were enrolled in this trial (Table 2). Dogs were assigned to the treatment with Tel-eVax based on owner’s consent. The group included 12% intact males, 47% neutered males, and 41% neutered females. Breeds included Golden Retriever (4), Giant Schnauzer (1), German Shepherd (2), Terrier Mix (1), Bassett Hound (1), English Bulldog (1), Springer Spaniel (1), Labradoodle (1), Pomeranian (1), Shih-tzu (1), Havanese (1) and mixed-breed dogs (2). The median age was 84 (range 39–158) months. The median weight was 36 (range 5.9–45.0) kilograms. All subjects were affected by DLBCL. The lymph node involvement was generalized with different prevalent locations. Selected animals did not show any additional significant disease. Only patient #6902 developed an Hemangiosarcoma (HSA) 9 months after BLBCL diagnosis. All the dogs included in the study received a l-CHOP 27-weeks protocol. Patients coded 3732, 1301, 5767 and 6902 received additional chemotherapy. Vaccine began at week 4 at the same date of Doxorubicin administration when most dogs (> 50%) achieved complete remission. Tel-eVax vaccination regimen is shown in Fig. 1a and consisted of two injections of Ad (10^11^ vp) in the biceps femoris at 2 weeks interval followed by one or more cycles of three DNA–EGT (5 mg/injection) every 2 weeks in the *tibialis cranialis* muscle with a Vet-ePorator™ device (Fig. 1b). An 8-needle array electrode was utilized in the area following injection with DNA to ensure full coverage by the electrical field and appropriate tissue electroporation (Fig. 1c). Most of the dogs received the complete treatment consisting of two Ad and 1 DNA–EGT cycle (Table 2). The profile of electroporation parameters was checked at every DNA administration in real time and data were stored into the Vet-ePorator™ archive for each single patient.

**Table 2:** Demographics

ID	Breed	Weight (kg)	Gender	Age (m)	Tumor type	Previous treatment	Number of boosters
3732	G. Schnauzer	40.7	MN	84	DLBCL	l-CHOP, bendamustine, epirubicin	None
1301	GR	37.5	FS	84	DLBCL	l-CHOP, mito/actinomycin, MOPP	None
4603	GR	37	M	93	DLBCL	l-CHOP	5
5767	GSD	44.4	M	60	DLBCL	l-CHOP, MOPP, GS-9219	None
5971	Mix K9	37	MN	141	DLBCL	l-CHOP	None
6042	Terrier Mix	33.2	MN	45	DLBCL	CHOP-MA	None
6355	GR	36.7	MN	132	DLBCL	l-CHOP	None
6377	Bassett	22.6	FS	108	DLBCL	l-CHOP	None
6902	GSD	40	MN	75	DLBCL, HSA	l-CHOP, leukeran	None
7402	Mix K9	26.7	FS	132	DLBCL	l-CHOP	4
7795	Springer Spaniel	16.3	MN	158	DLBCL	l-CHOP	None
138	Golden Ret	45.1	FS	52	DLBCL	l-CHOP	None
387	Labradoodle	34.7	MN	63	DLBCL	l-CHOP	3
457	Pomeranian	5.9	FS	49	Stage V BLSA (bone marrow)	l-CHOP	None
477	Shihtzu	6.5	MN	72	DLBCL	l-CHOP	None
492	Havanese	22.9	FS	87	DLBCL	CHOP	2
1108	GR	38.6	FS	39	DLBCL	l-CHOP	None

*MN* male neutered, *FS* female spayed, *M* male, *HSA* hemangiosarcoma, *BLSA* B cell lymphosarcoma, *MOPP* (M)ustargen (O)ncovin + (P)rocarbazine + (P)rednisone

### Tel-eVax does not show side effects

To assess potential side effects connected with the immunotherapy, body weight and temperature were measured throughout the entire course of the study. No significant changes were noticed during the entire course of the study (not shown). To monitor signs of toxicity and/or to detect indications of autoimmunity, vaccinated animals were constantly monitored for abnormal values in hematological parameters. No significant hematological side effects connected with the immunizations were detectable in the treated animals.

### Tel-eVax induced Antibody responses

**Fig. 2:**
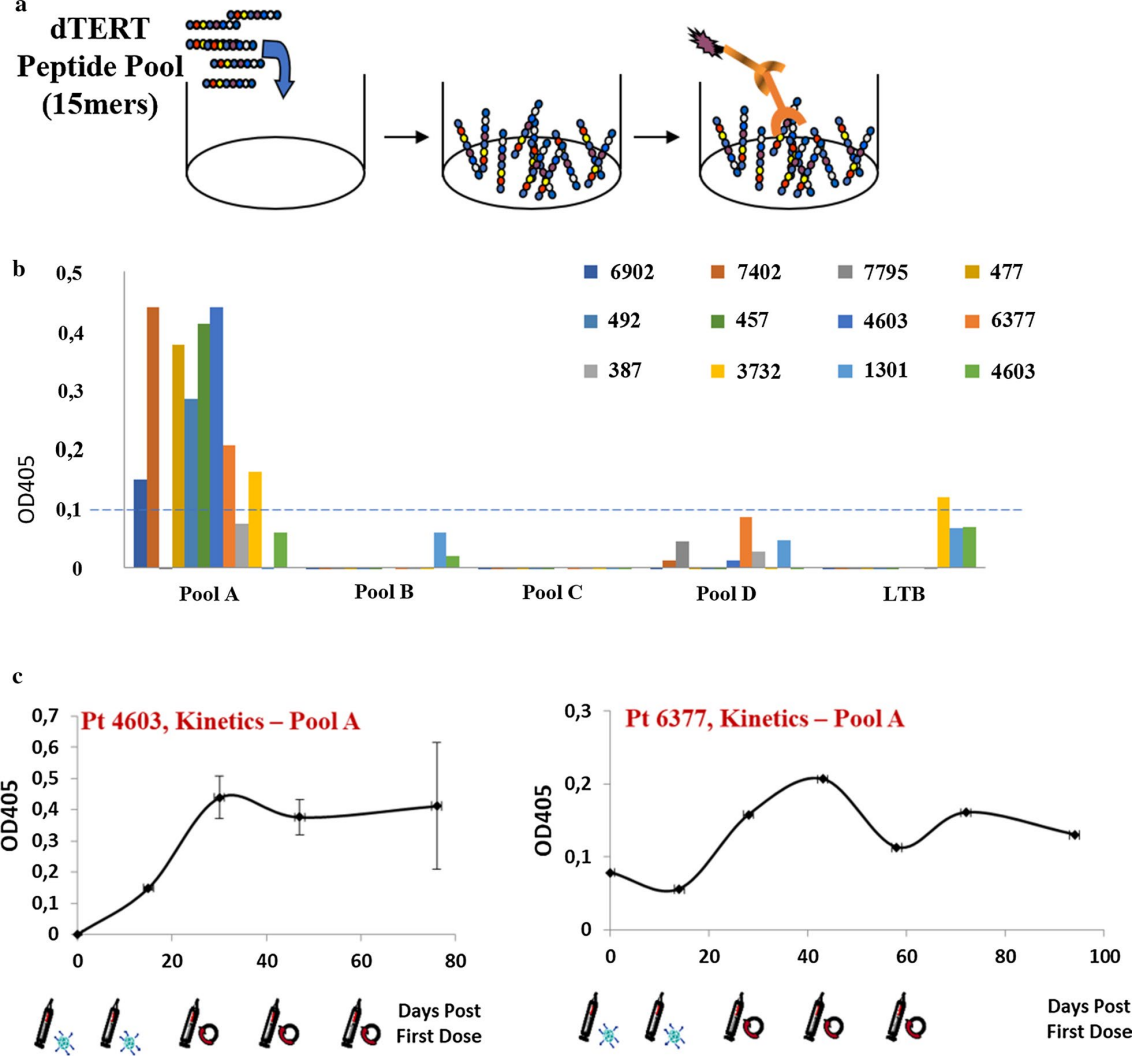
Antibody detection and kinetics. **a** representation of the ELISA assay. Peptides are coated on the bottom of plastic wells. Incubation with dog sera and detection with a AP-conjugated anti-canine IgG allow signal detection. **b** Antibody detection in 12 dogs. An arbitrary threshold ∆OD_405_ > 0.1 was used to identify responders. **c** Patient 4603 and patient 6377 antibody kinetics over time

Telomerase reverse transcriptase is a ribonuclear protein, however the immunogen was engineered as a fusion with Tissue Plasminogen Activator (TPA) leader sequence that makes it secreted by muscle cells following DNA–EGT [35]. Therefore, antibodies may represent the easier surrogate biomarkers to monitor the immune response in vaccinated dogs. On this purpose, we sought to follow antibodies against TERT and against the non-self-part of the immunogen, *Heat labile enterotoxin* B (LTB). Since TERT protein is not available, we used 4 pools of 15mer peptides overlapping by 11 residues and covering the entire dTERT protein. Peptides were coated onto ELISA plates (Fig. 2a) and dog serum was incubated at different dilution to measure antibodies. At day 45 post priming, sera from 12 dogs were analyzed by ELISA. We arbitrarily assumed as a threshold a signal ∆OD_405_ > 0.1 compared with the DMSO control. As shown in Fig. 2b, most of the dogs (8 out of 12) developed a low but detectable seroconversion against Pool A, which covers the N-terminal of TERT protein. Poor/no signal was measured against Pool B, C, D and LTB. In two dogs (Pt4603 and Pt6377) we were able to follow antibody kinetics over time (Fig. 2c). These data indicate that dTERT was expressed in vivo and that antibodies were induced upon Tel-eVax™ treatment.

### Tel-eVax prolongs survival of CHOP-treated canine DLBCL

**Fig. 3:**
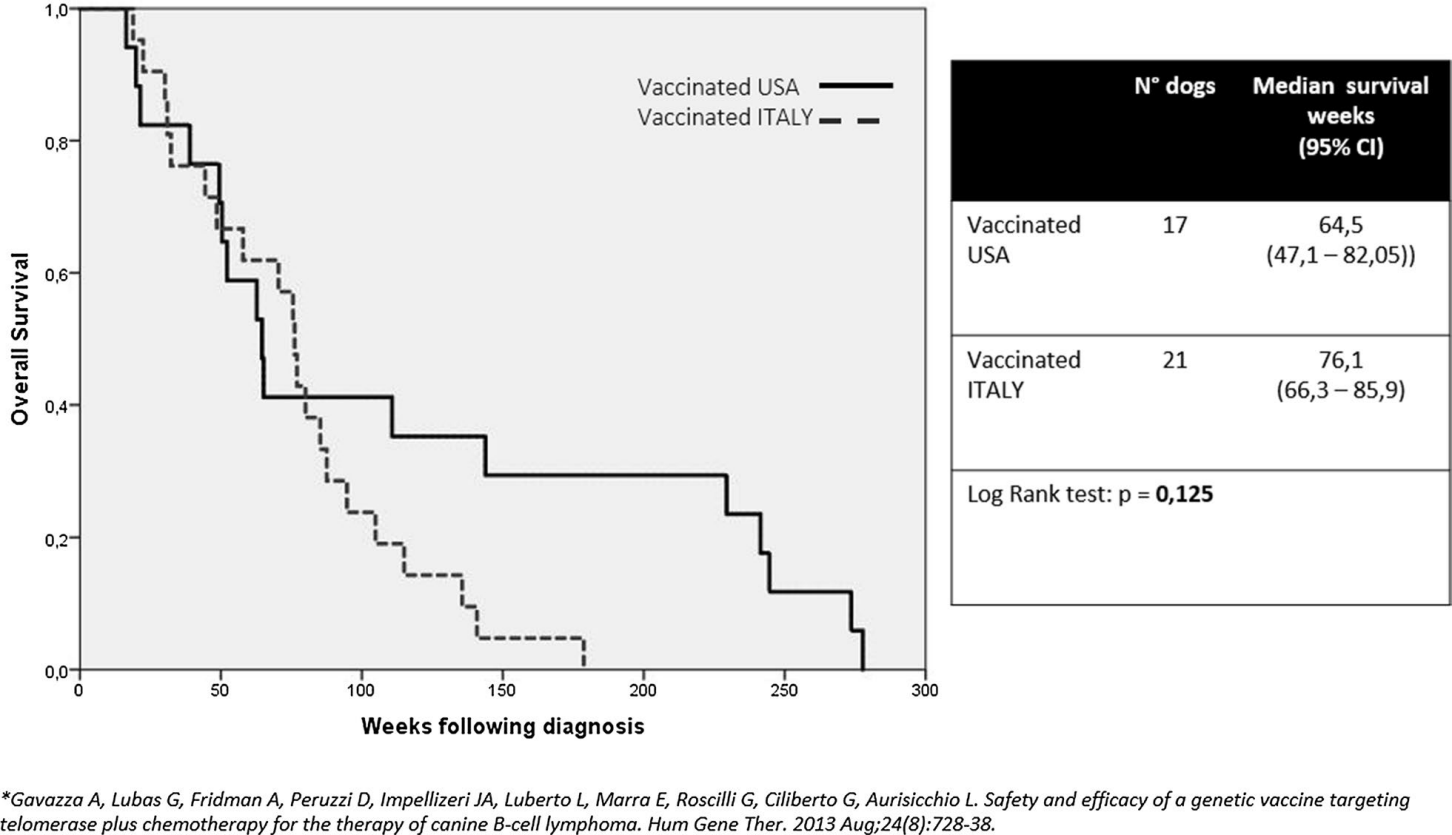
Kaplan–Meier estimates. Overall Survival in two Lymphoma cohorts treated with Tel-eVax™/Chemotherapy: vaccinated USA (this study) and vaccinated Italy [28]. Median survival weeks are shown

Tel-eVax/CHOP treated DLBCL dogs have been monitored over time for overall survival (OS). In the monitored period (277 weeks), there have been 94% deaths in the group, mostly attributable to DLBCL relapse. The 95% lower confidence interval for OS of the Tel-eVax/CHOP group was 452 days, corresponding to 64.5 weeks. There was no observable difference in survival both by gender and age. The evaluations included all Tel-eVax/CHOP dogs initiating treatment, irrespective of reason for not completing the study. Figure 3 compares the OS observed in this study with the Tel-eVax/COP combination described in Gavazza’s study [34]. Interestingly, no significant difference between the two cohorts was observed (log-rank test, p = 0.125), confirming the beneficial effect of Tel-eVax™ in addition to chemotherapy for DLBCL and increased OS.

## Discussion

Lymphoma is the most common hematopoietic tumor in dogs. Approximately, 80% are B immunophenotype [36]. The standard of care treatment comprises chemotherapy with COP or CHOP as the most common protocols and often based on user comfort and financial limitations of the client. COP chemotherapy regimen can be considered less effective, as CHOP has shown superior efficacy [37]. CHOP consists of a 15–27-week induction protocol including an alternating schedule of cyclophosphamide, doxorubicin, vincristine and prednisone with or without l-asparaginase. Regardless, median survival for canine patients is between 275 and 397 days [4–7].

In two previous studies [33, 34], we have shown that Tel-eVax is safe, immunogenic and most importantly had a significant therapeutic impact on DLBCL dogs when combined with COP. dTERT-specific cell mediated immune responses were induced in almost all treated animals. No adverse effects were observed in any canine patient that underwent treatment. In the double arm study, the OS time of vaccine/COP treated dogs was significantly increased over the COP-only treated cohort (> 76.1 vs 29.3 weeks, respectively, p < 0.0001). An open question in our previous study was the possibility that COP maintenance chemotherapy may act as a immune-modulator in the vaccination protocol. Some classes of chemotherapy drugs may indeed affect antigen cross-presentation [38], induce a cytokine storm [39], reduce the number of regulatory T cells [40, 41], and activate homeostatic lymphoid proliferation [42] that could help induce the immune response against self-antigens [43]. TERT is an interesting and safe immunologic target as it is overexpressed mainly by cancer cells. In our experience with TERT-immunotherapies we have observed autoimmunity effects marked by a consistent, although transient, B-cell depletion after TERT-specific adoptive T cell transfer [44] in a rodent model but never with a genetic vaccination approach.

In this study, we have combined Tel-eVax with CHOP, a dose-intense chemotherapy regimen applied by most veterinary oncologists in the United States. The primary end point of the study was to assess the efficacy of the combination on progression and evolution of canine DLBCL overall survival time. No adverse effects were observed in any patient, such as significant changes in hematological parameters (not shown), or adverse clinical signs, as also not reported by the owners. To further follow the immune response in these patients, we have tested a novel immunologic assay aiming at detecting anti-dTERT antibodies in vaccinated patients’ serum. In the absence of the full-length protein, we used pools of 15mer peptides covering the antigen as a tool to detect antibodies. Most of the tested dogs (8 out of 12) mounted a low but detectable antibody response (Fig. 2) against Pool A, corresponding to the N-terminus of the protein. A potential reason may be the presence of B cell epitopes present in that pool and exposed on folded protein upon secretion. Strikingly, we have not observed antibodies against LTB, the non-self-portion of the recombinant protein expressed by Tel-eVax. There are two possible explanations for these findings: (1) TPA-dTERT-LTB fusion protein is not correctly folded and despite its immune-enhancing property, LTB is masked to the immune system. This observation is corroborated by the low cell mediated immune response against LTB peptides in different TERT fusions in mice [45], dogs [33, 34], non-human primates [46] and in a Phase I clinical trial in human cancer patients (manuscript in preparation) whereas it did not happen with a different fusion partner such as Carcinoembryonic Antigen [47]; (2) since it was done with peptides, the assay can only detect linear but not conformational epitopes. Therefore, we cannot exclude the presence of other antibodies elicited against TERT or LTB. The availability of the recombinant protein or its domains may therefore be instrumental for the optimization of the immune assay.

Finally, a significant therapeutic impact on survival time was observed in the Tel-eVax/CHOP group (Fig. 3). The absence of control arm is of course a limitation of our study and therefore we cannot determine whether the nature of the combination is synergistic and additive. However, we know that the vaccine without chemotherapy does not work [33]. This may be due the fact that the immune response takes time to get induced and work in a minimal residual disease setting. At any rate, the overall survival of animals was 452 days, which is far beyond the historical survival data reported in literature with CHOP regimens. On this basis, a potentiation effect over chemotherapy-only seems to be the nature of such combination. Moreover, since the study was conducted in New York state, we have compared our survival data with current data relative to Northern United States for DLBCL. An indirect comparison between the 17 patients treated in this study with the dog cohort of lymphoma patients described in Wilson-Robles et al. (452 and 244 days, respectively) suggest a ~ 2-fold OS increase, in line with our previous findings. In addition, no statistical difference (p = 0.125) was observed between Tel-eVax/CHOP and Tel-eVax/COP data generated in our previous two studies, thus confirming the efficacy of Tel-eVax in DLBCL. Of note, the minimal effective dose of Tel-eVax in dogs has not been yet identified: we used 10^11^ vp Ad or 5 mg DNA plasmid/administration in our three studies but we cannot exclude similar antitumor effects with lower, saturating doses.

Recently, a conditionally licensed treatment for canine lymphoma, Rabacfosadine, has shown substantial single-agent activity in dogs with lymphoma [48]. Rabacfosadine treatment resulted in overall response rate 84% (68% Complete response, CR; 16% partial response, PR with an overall median progression‐free interval (PFI) of 194 days (216 for CR and 63 for PR). Combining and/or comparing Tel-eVax with rabacfosadine in a future clinical trial would be of great interest. More importantly, Tel-eVax concept (Ad + DNA-EP targeting hTERT) can be applied to human patients. Multiple mechanisms have in fact been shown to increase Telomerase activity in hematological malignancies (e.g. epigenetic modulation, amplification of the *hTERT* gene), thus suggesting that TERT may represent an optimal target for human DLBCL [25].

## Conclusions

In summary, our study shows that Tel-eVax is safe and has a significant impact on DLBCL canine patients’ survival when combined with CHOP chemotherapy. Our data further supports the clinical use of Tel-eVax in multicentric DLBCL with application towards conditional licensure and warrants the evaluation of this treatment in other canine tumor types. Of note, given the increasing importance of the dog as translational model for human diseases, our data are promising for the evaluation of hTERT-targeting immunotherapy for human hematological malignancies.

## Abbreviations

Ad: Adenovirus
EGT: Electro-Gene-Transfer
TERT: telomerase reverse transcriptase
COP: cyclophosphamide, vincristine, prednisone
CHOP: cyclophosphamide, vincristine, prednisone, doxorubicin
